# A novel heterozygous splice-altering mutation in *HFM1* may be a cause of premature ovarian insufficiency

**DOI:** 10.1186/s13048-019-0537-x

**Published:** 2019-07-06

**Authors:** Jing Zhe, Shiling Chen, Xin Chen, Yudong Liu, Ying Li, Xingyu Zhou, Jun Zhang

**Affiliations:** Center for Reproductive Medicine, Department of Obstetrics and Gynecology, Nanfang Hospital, Southern Medical University, Guangzhou, 510515 People’s Republic of China

**Keywords:** Premature ovarian insufficiency, *HFM1*, Whole-exome sequencing, Splicing minigene

## Abstract

**Background:**

Premature ovarian insufficiency (POI) leads to early loss of ovarian function in women aged < 40 years and is highly heterogeneous in etiology. The genetic etiology of this disorder remains unknown in most women with POI.

**Methods:**

Whole-exome sequencing (WES) was used to analyze genetic factors within a Chinese POI pedigree. Bioinformatic analysis was applied to identify the potential genetic cause, and Sanger sequencing confirmed the existence of a mutation within the pedigree. A minigene assay was performed to validate the effect of the mutation on pre-mRNA splicing.

**Results:**

A novel heterozygous missense mutation in *HFM1* (c.3470G > A) associated with POI was identified by whole-exome sequencing. This mutation was heterozygous in the affected family members and was absent in the unaffected family members. In silico analysis predicted that the mutation was potentially pathogenic. Bioinformatic splice prediction tools revealed that the mutation was very likely to have a strong impact on splice site function. Results of the minigene assay revealed that the mutation changed the mRNA splicing repertory.

**Conclusions:**

The missense mutation of the *HFM1* gene (c.3470G > A) may be a cause of POI. The mutation altered mRNA splicing in cells. This study can provide geneticists with deeper insight into the pathogenesis of POI and aid clinicians in making early diagnoses in affected women.

## Background

Premature ovarian insufficiency (POI) is a clinical syndrome defined by loss of ovarian activity before the age of 40 and is characterized by amenorrhea or oligomenorrhea for at least 4 months and raised serum follicle-stimulating hormone (FSH) levels > 25 IU/L on two occasions > 4 weeks apart [1]. POI has a great impact on women’s fertility and long-term health [2]. It affects approximately 1 in 100 women and 1 in 1000 women at the ages of 40 and 30, respectively [3]. Women with POI have menopausal symptoms such as hot flashes, sleep disturbance, vaginal dryness and emotional disturbance in the short term and increased risk of cardiovascular disease, Alzheimer’s disease and osteoporosis in long term [4–6].

POI is highly heterogeneous in etiology, which includes genetic, iatrogenic, autoimmune, and infectious factors [7]. Although the disorder is generally considered to have a strong genetic link, the underlying cause remains unknown in many women with POI [4]. With a decrease in running costs, next-generation sequencing (NGS) has recently been widely used in the field of research and clinical medicine and has revealed genetic causes in 20–25% of women with POI [8, 9]. Furthermore, genetic predisposition can be verified by analysis of familial aggregation of women with POI, suggesting existence of inherited genetic defects in these families [10].

In this study, we used whole-exome sequencing (WES) to evaluate the genetic cause of POI in a Chinese family with a family history of POI. A minigene splicing assay was performed to identify a novel heterozygous splice-altering mutation in the meiotic-related gene *HFM1*. To the best of our knowledge, this study is the first to report the mutation of the *HFM1* gene (c.3470G > A) as a potential cause of POI. The contribution of the mutation to the etiology of POI was evaluated.

## Methods

### Study participants and their families

A Han Chinese family was recruited in Nanfang Hospital, Guangzhou, Guangdong, China. POI was diagnosed in accordance with previously described criteria [1], that is, a depletion or loss of normal ovarian function in women before 40 years of age with FSH > 25 IU/L on two occasions > 4 weeks apart. The proband (II-1) and her mother (I-1) were affected by POI at the ages of 31 and 30 years old, respectively. The proband’s sister(II-2)was fertile and had no difficulty conceiving. The Ethics Committee of Nanfang Hospital, Nanfang Medical University (NFEC-2017-197) approved this study, and written informed consent was obtained from all participants.

### DNA extraction and WES

Total genomic DNA was extracted from all participants by using a TIANamp Blood DNA Kit (Tiangen, Beijing, China) according to the manufacturer’s protocol. Samples from the proband, her sister and their parents were subjected to WES using a HiSeq Xten sequencing platform (Illumina, San Diego, California, USA). The raw sequencing data included 58,850,887,060 reads, and the average raw sequencing depth (X) was 139.32.

### Mutation validation and co-segregation analysis

Sanger sequencing was performed using specific PCR primers designed with Primer Premier 5 (http://www.premierbiosoft.com/primerdesign/). The sequences of *HFM1* primers used were *HFM1*-F: 5′-TTCATGTTGCCCACAGAGAGAA-3′ and *HFM1*-R: 5′-TTGTCTGAAAGGAAGGAAACTGG-3′. PCR amplification included 1 cycle at 98 °C for 30 s followed by 35 cycles at 98 °C for 5 s, 55 °C for 5 s, and 72 °C for 20 s per 1 kb as an extension step. A final extension step of 1 cycle at 72 °C for 60 s was also performed. The *HFM1* mutation was validated as previously described, and segregation analysis was performed in the family members.

### In silico prediction

The potential pathogenicity of the mutation was predicted by in silico analysis using four different tools: Polyphen-2 (http://genetics.bwh.harvard.edu/pph2), MutationTaster (http://www.mutationtaster.org/), SIFT (http://sift.bii.a-star.edu.sg/) and Combined Annotation Dependent Depletion (CADD, https://cadd.gs.washington.edu/). The potential effect on splicing was predicted with two splice-site prediction programs, i.e., Human Splicing Finder (HSF, http://www.umd.be/HSF3/HSF.shtml) and Splice Site Prediction by Network (http://www.fruitfly.org/seq_tools/splice.html).

### Minigene splicing assay

#### Synthesis of hybrid minigenes

A minigene splicing assay was performed to verify whether the mutation affected splicing products. To test the effect of the candidate mutation c.3470 G > A in *HFM1* on splicing, amplicons generated by standard overlapping PCR and digestion procedures were cloned into the pcDNA3.1 reporter vector (Life Technologies, New York, USA). PCR and Sanger sequencing were used to evaluate whether the wild-type (WT) and mutant-type (MT) expression vectors had been successfully constructed.

#### Characterization of *HFM1* expression

WT or MT *HFM1* minigenes were transiently transfected into HeLa cells and 293 T cells using Liposomal Transfection Reagent(40802ES03, YEASEN, Shanghai, China) according to the manufacturer’s instructions.

All cellular RNAs were extracted using the RNAiso Plus (Code No.9109, Takara, Otsu, Japan) according to the manufacturer’s recommendation. Total RNA was used to produce cDNA using the PrimeScript RT reagent Kit with gDNA Eraser (Cat#RR047A, Takara, Otsu, Japan). Then, the first-strand cDNA was synthesized and amplified. The PCR products were separated and verified by electrophoresis in an agarose gel and characterized by direct Sanger sequencing.

## Results

### Clinical findings

**Fig. 1:**
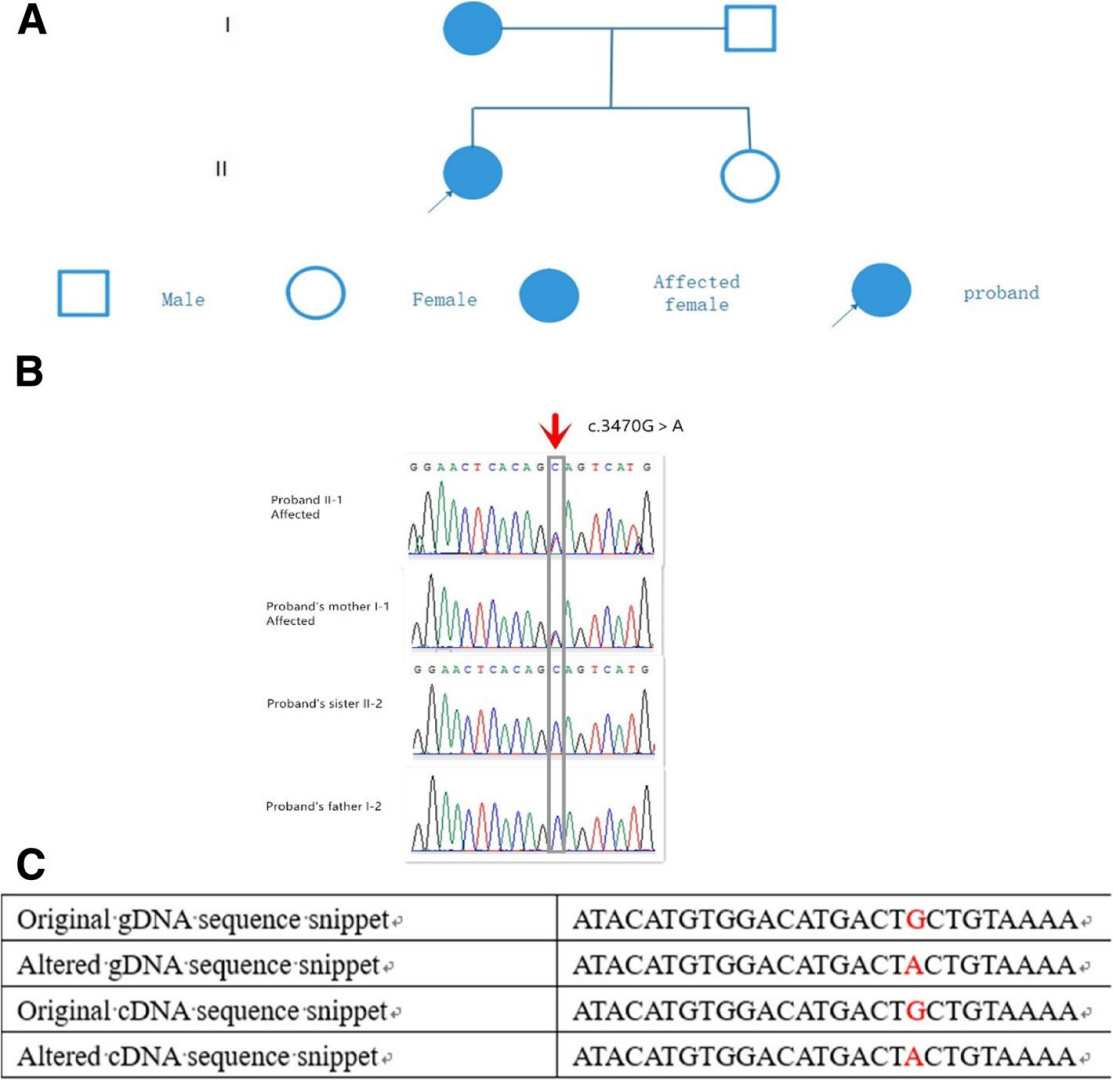
Pedigree and genetic analysis of the Chinese family. (**a**) Two family members in this pedigree were diagnosed with POI. The solid circle with an arrow indicates the proband. Solid circles indicate the affected family members. (**b**) Validation of the mutation by Sanger sequencing. The red arrow and gray frame indicate the mutation site (c.3470G > A) in I-1 and II-1 as double peaks and the lack of mutation in I-2 and II-2 as a single peak. (**c**) Original and altered gDNA and cDNA sequence snippets. The location of c.3470G>A is marked in red

The proband (II-1) and her mother (I-1) were diagnosed with POI (Fig. 1a). The proband, a 33-year-old woman, had been diagnosed at the age of 31. She experienced menarche at the age of 14 and had 28–30 day menstrual cycle but had irregular menstrual cycles after 2015. She had a termination of pregnancy with an unplanned natural pregnancy at 31 years old, then was diagnosed with POI in the same year. She had a natural pregnancy at the age of 32, but unfortunately the embryo stopped to develop at 8 weeks. Her basic hormone levels were as follows: FSH, 78 mIU/ml; luteinizing hormone(LH), 31 mIU/ml; estradiol(E2), 13 pg/ml; prolactin(PRL), 13 ng/ml; testosterone(T), 18 ng/dl; and anti-Mullerian hormone (AMH), 0.06 ng/ml, respectively with the examination in Nanfang Hospital, Guangzhou. Transvaginal ultrasound examination revealed a normal-sized uterus (37 × 35 × 32 mm). The endometrium was 2.5 mm in thickness. The sizes of the two ovaries were smaller than that of a normal ovary (left ovary: 11 × 8 × 10 mm; right ovary: 15 × 7 × 12 mm), and only one antral follicle was observed in the left ovary.

The proband’s mother (I-1) experienced menarche at 13 years old, which was within the normal age range. She had an early marriage and suddenly underwent menopause a year after the birth of her last child and was diagnosed with POI at the age of 30. Both the proband and her mother had normal 46, XX karyotypes with normal height, weight and external genital organs. Neither woman had a history of relevant surgeries, endocrinopathies or autoimmune disorders. The fertile younger sister, aged 30 years old, had normal spontaneous pubertal development and conceived spontaneously and delivered a healthy girl.

### WES analysis and *HFM1* mutation co-segregation with POI in this family

WES was performed on the proband, her sister and their parents. Harmful mutations were identified by screening the mutation sites. First, mutation sites with population frequencies > 0.01 were filtered from the 1000 Genomes Project database. Next, mutation sites with frequencies > 0.01 were filtered from the Exome Aggregation Consortium database (ExAC) and the Exome Sequencing Project 6500 (ESP6500) dataset. Third, mutations in exonic regions or splicing regions were preserved. Fourth, synonymous mutations were filtered out, and nonsynonymous mutations were retained. Finally, we used four different tools to predict the potential pathogenicity of the identified mutations.

**Fig. 2:**
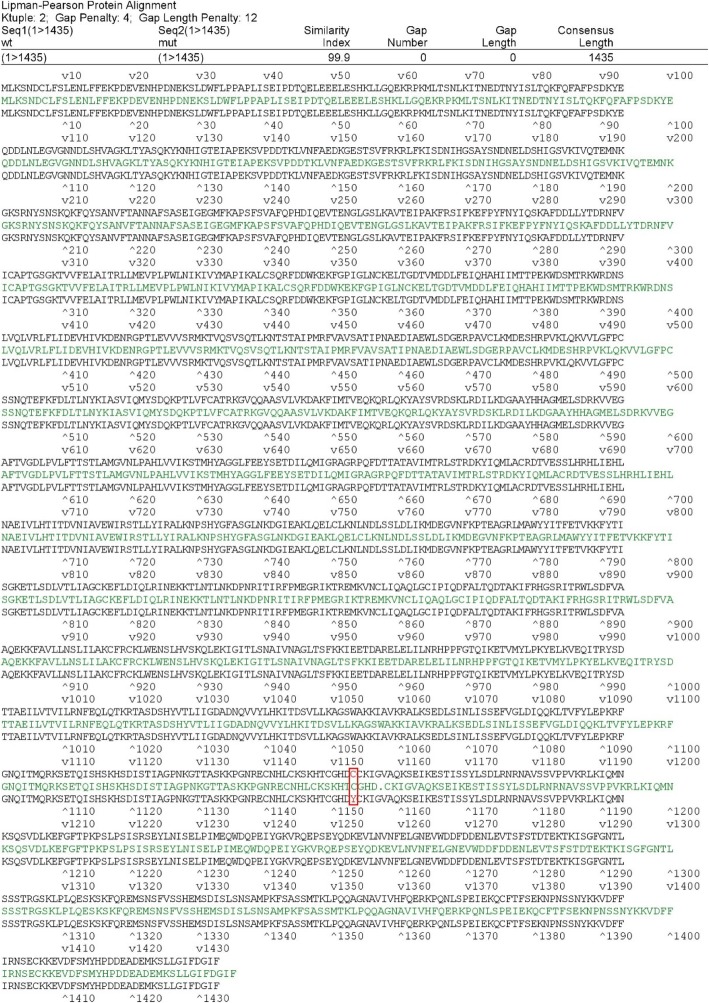
Comparison of wild-type and mutant amino acid sequences. The red residues in the position marked with a red box are the amino acids at the *HFM1* mutation site

Based on the pipeline output and the several filtering steps after WES, a heterozygous missense mutation in the ATP-dependent DNA helicase homolog (*HFM1*), namely, NM_001017975:exon31:c.3470G > A, was identified in the proband and her mother (Table 1). The original and altered gDNA and cDNA sequence snippets are shown in Fig. 1c. Missense mutation caused changes in the amino acid sequence from the WT amino acid sequence (Fig. 2).

**Table 1:** In silico analysis of the *HFM1* mutation

Gene	Mutation	Amino acid change	Zygosity	SIFT score	SIFT_pred	Polyphen2_HDIV_score	Polyphen2_HDIV_pred	Polyphen2_HVAR_score	Polyphen2_HVAR_pred	MutationTaster_score	MutationTaster_pred	CADD_phred
HFM1	c.3470G>A	G > A	Heterozygous	0	D	1	D	0.998	D	1	D	29.7

“D” indicates that the mutation is damaging or deleterious. In SIFT (http://sift.bii.a-star.edu.sg/), mutations with values above 0.05 are predicted to be damaging. In Polyphen-2 (http://genetics.bwh.harvard.edu/pph2), higher scores predict the mutation to be more deleterious. In MutationTaster (http://www.mutationtaster.org/), the probability value ranges from 0 to 1, and a higher score (closer to 1) indicates a stronger prediction of disease. In Combined Annotation Dependent Depletion (CADD) (http://cadd.gs.washington.edu/), the suggested threshold for harmfulness of a single-nucleotide polymorphism (SNP) is a CADD_Phred score > 15

Sanger sequencing was performed to validate the *HFM1* mutation shared by the two POI patients in the family, and Mendelian segregation in the family members was tested. The proband (II-1) and her mother (I-1) had the heterozygous mutation in *HFM1*, whereas the father (I-2) and the unaffected sister (II-2) did not have the mutation (Fig. 1b).

### Potential effects of the *HFM1* mutation

**Fig. 3:**
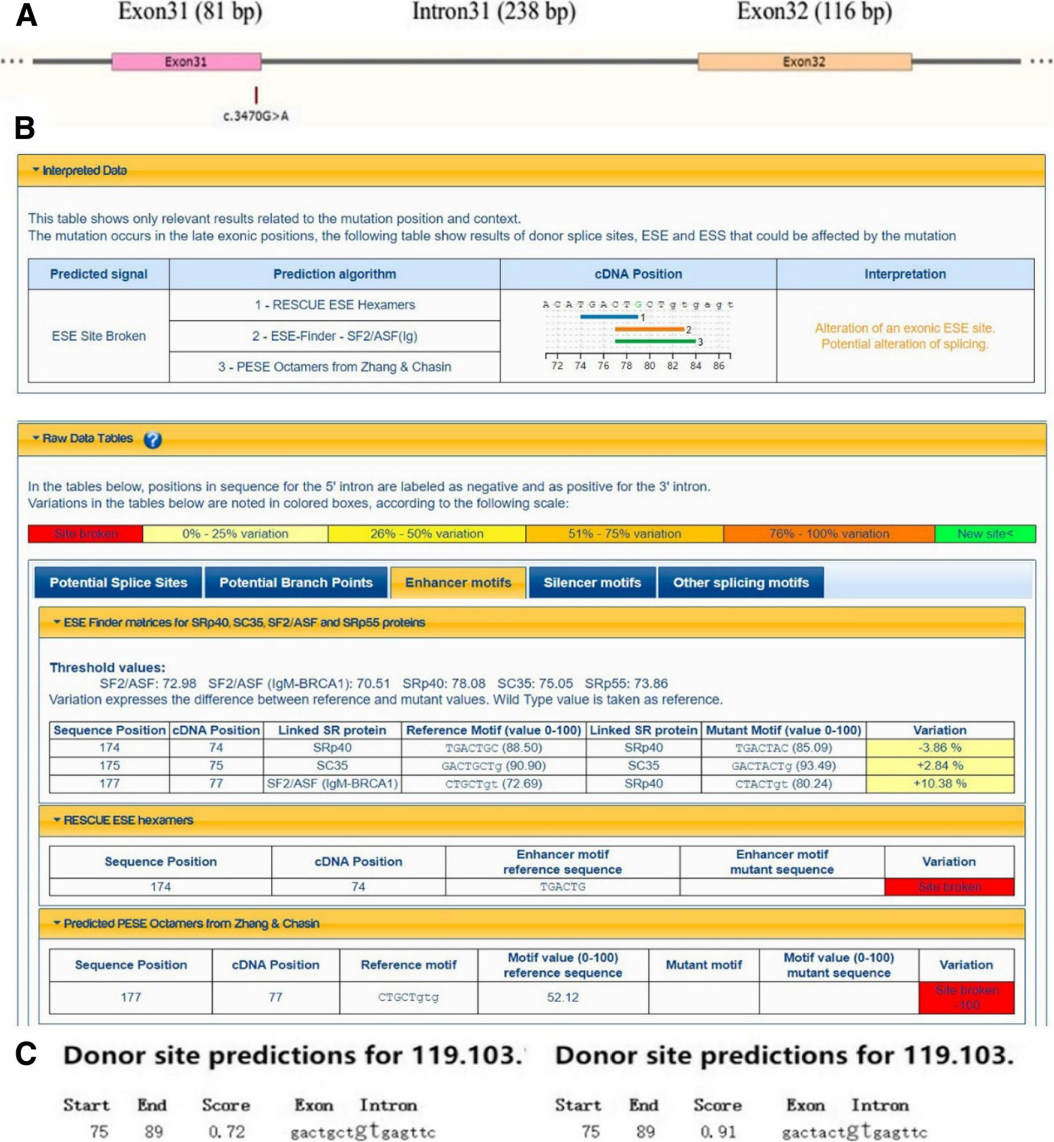
Analysis of potential functions of the *HFM1* mutation. (**a**) Schematic diagram of the location of the *HFM1* mutation with regard to exons and introns. The missense mutation in *HFM1* (c.3470G > A) is located at the third-to-last base of exon 31. (**b**) The underlined sequences were analyzed, and possible changes in splice sites were predicted with HSF. (**c**) Differences in confidence scores before vs. after mutation as determined with Splice Site Prediction by Neural Network

The identified missense mutation in *HFM1* (c.3470G > A) was located on the third-to-last base of exon 31 (Fig. 3a). This location was very close to an intersection of splice sites and could thus affect the splice sites. Therefore, we used Human Splicing Finder (HSF) and Splice Site Prediction by Neural Network to predict changes in splice sites caused by the mutation. The first prediction tool predicted that the mutation could potentially alter splicing, possibly causing changes or destruction of splicing enhancer sites and resulting in splice site abnormalities (Fig. 3b). According to the second splice prediction tool, the confidence scores, which predict the possibility of selective splicing, were 0.72 to 0.91 before and after mutation, respectively (Fig. 3c). The increasing score indicated that it was likely to be affected splice site. In summary, both splicing prediction software programs indicated that the mutation (c.3470G > A) was likely to influence splicing products.

### Results of the Minigene assay

#### Colony PCR test and sequencing in recombinant vector

**Fig. 4:**
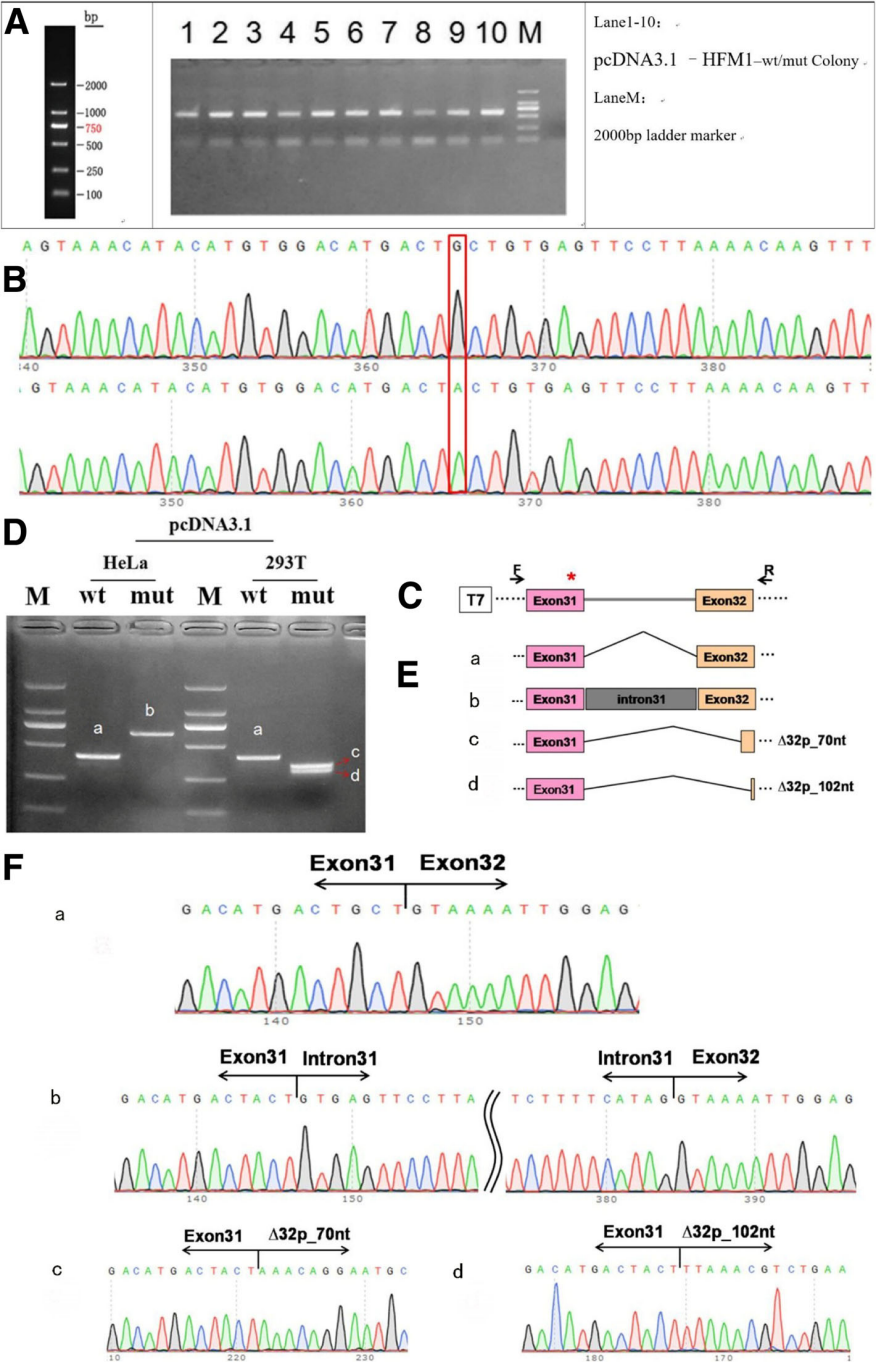
Splicing alteration was identified by a minigene assay. (**a**) Amplification bands of colony PCR tests in pcDNA3.1-wt/mut. (**b**) Sequencing in the recombinant vector. The top of Fig. 4b indicates the results of WT minigene sequencing, and the bottom shows the sequencing of the MT (c.3470G > A) minigene. Both of them are partial sequencing results. The red frame indicates the base changed by the mutation. (**c**) Schematic diagram of minigene construction. (**d**) Reverse-transcription polymerase chain reaction (RT-PCR) products were separated by electrophoresis of the pcDNA3.1 vector in HeLa and c293T cells. (**e**) Schematic diagram of Sanger sequencing of RT-PCR products. (**f**) The sequencing results for the bands

The colony PCR tests in pcDNA3.1-wt/mut were done with the following primers: pcDNA3.1-F:5′-ACTTAAGCTTATGGGAAC-3′ and pcDNA3.1-R:5′-CGGTGGATCCCTTCAGACGTTTAACTGGAGGA-3′. The amplified bands (Fig. 4a) were cloned, and the resulting clones were sequenced. The sequencing results indicated that the Wild type(WT) and Mutant type (MT) minigenes were inserted into the pcDNA3.1 vector successfully. The sequence difference between before and after the mutation is shown in Fig. 4b.

#### RNA extraction and cDNA for RT-PCR

RNA was extracted and reverse-transcribed into cDNA after plasmid transfection of cells for 48 h. Total RNAs were qualified for each sample, with an average mass of 402 ng and integrity of 1.879.

#### Transfection and quantitative analysis of transcript mutation

To verify the effect of the c.3470G > A mutation in *HFM1* on pre-mRNA splicing, we performed a minigene assay. In the assay, a mutant *HFM1* minigene was transfected into HeLa cells and 293 T cells. A total of 4 samples were collected after 36 h of transfection. A schematic diagram of the minigene construct is shown in Fig. 4c.

The results of RT-PCR showed that there was only one WT and one MT band in HeLa cells. The WT band with the expected size was named band a, while the MT band was named band b (Fig. 4d). Sequencing showed that the WT band was cleaved normally (400 bp), i.e., the cleavage mode of the band was Exon 31 (81 bp)-Exon 32 (116 bp) (Fig. 4E-a). The MT band b (638 bp) retained all the intron 31. The cleavage mode of the band b was Exon31-intron31-Exon 32 (Fig. 4E-b). In the 293 T cells, the minigene splicing assay of the WT and MT constructs revealed one expected band produced by WT (a) and two bands of similar size in MT. The large band and the small band were named c and d respectively (Fig. 4d). Sequencing of each band showed that band a (400 bp) had normal splicing, and its cleavage mode was Exon 31 (81 bp)-Exon 32 (116 bp), as above. Band c showed a deletion of 70 bp on the left side of exon 32, i.e., the cleavage mode was Exon 31 (81 bp)-ΔExon 32 (46 bp) (Fig. 4E-c). Band d included a deletion of 102 bp on the left side of exon 32. Its cleavage mode was Exon 31 (81 bp)-∆Exon 32 (14 bp) (Fig. 4E-d). The sequencing results of the bands are shown in Fig. 4f. These results show that the *HFM1* mutant minigenes produced alternative transcripts different from the WT minigene in two cell types.

## Discussion

In this pedigree, we identified a novel missense mutation c.3470G > A in *HFM1* associated with POI. Our study showed that the mutation altered mRNA splicing. The proband and her mother were heterozygous carriers of the mutation and exhibited the POI phenotype, whereas the sister and the father of the proband did not have the mutation.

*HFM1* is a gene involved in meiosis, which is a critical process in generating haploid gametes. It is a specialized type of nuclear division [11, 12], and genes participating in meiosis are potential candidates in POI pathogenesis [13, 14]. Most mutations in genes associated with POI have deleterious impacts on the germ cell pool [15].

*HFM1*, comprising 39 exons mapped to human chromosome 1q22, is a meiosis-specific gene and is expressed in germline tissues [16–18]. *HFM1* encodes a DNA helicase essential for meiotic homologous recombination. Its transcript is preferentially expressed in ovaries [19]. One mutation in *HFM1* has been found to reduce crossover frequency and impair double-strand break repair, resulting in changes to the DNA during meiotic recombination [20]. Guiraldelli et al. reported that *HFM1-*deficient mice were infertile [21]. *HFM1*-knockout mice exhibit phenotypes resembling POI in humans. Recent evidence suggests that mutations in *HFM1* could cause POI in humans, as compound heterozygous mutations in the *HFM1* gene were identified in two affected POI sisters and a woman with sporadic POI [22]. In the former case, both parents were clinically normal and carried one of the mutations, whereas the two affected sisters had the same compound heterozygous mutation in *HFM1*. These findings demonstrate that *HFM1* mutations are etiologic factors of POI at the genetic level.

It is reported that *HFM1* mutation is as a cause of POI as a previous report in a Chinese pedigree. [22]. In our case, we found that the POI pedigree represents that the proband and her mother with POI had this mutation in *HFM1* whereas the unaffected sister and their father did not have one. We also found that both of the POI patients had a history of birth or pregnancies before or after the diagnose of POI. The reason why the differences may be due to incomplete penetrance or the different degrees of expressivity in individuals. In our study, the novel heterozygous splice-altering mutation in *HFM1*(c.3470G > A) may be a cause of premature ovarian insufficiency and further exploration can be made to explain the in-depth pathogenesis.

Pathogenic mutation can influence mRNA transcription. At the level of the pre-mRNA processing, splicing defects play a majority role in human diseases. The functioning of basic and auxiliary splicing elements is necessary for proper pre-mRNA splicing [23]. To date, a series of splice-site mutations are known. In Chinese POI patients, a splicing mutation (c.1686-1G > C) in *HFM1* has been reported in a previous study [22]. Moreover, Pu et al. studied 138 Chinese sporadic POI cases and identified 5 new missense mutations, but no functional studies were conducted [17]. In our study, since the missense mutation in *HFM1* (c.3470G > A) was very close to an intersection of splice sites, we used bioinformatic splice prediction tools to assess the possible effect of the c.3470G > A mutation, which revealed that the mutation might have a great impact on splice site function. It is reported that a minigene experiment can produce splicing results reaching almost 100% similarity [24]; therefore our minigene splicing assay of the WT and MT constructs revealed that an alternative splicing process were produced with the c.3470G > A mutation in mRNA level. Further studies are still needed to confirm the role of the *HFM1* gene mutation (c.3470G > A) in POI in protein level and potential functions in cells of involved in meiosis, which is a critical process in human fertility.

## Conclusions

In summary, we identified a novel heterozygous missense mutation in the *HFM1* gene (c.3470G > A) in two POI patients of a Chinese family by WES. The mutation of *HFM1* gene at c.3470G > A caused a splicing defect by in vitro minigene assay. It is suggested that the splice-altering mutation in *HFM1* is a likely cause of POI.

## Data Availability

All data generated or analyzed during this study are included in this published article.

## Abbreviations

AMH: Anti-Mullerian hormone
CADD: Combined Annotation Dependent Depletion
E2: Estradiol
ESP6500: Exome Sequencing Project 6500
ExAC: Exome Aggregation Consortium database
FSH: Follicle stimulating hormone
HSF: Human Splicing Finder
LH: Luteinizing hormone
MT: Mutant type
NGS: Next-generation sequencing
POI: Premature ovarian insufficiency
PRL: Prolactin
T: Testosterone
WES: Whole-exome sequencing
WT: Wild type
